# Prognostic Relevance of Ascites Volume and Dynamics in Outpatients with Cirrhosis: A Prospective Cohort Study

**DOI:** 10.3390/jcm15072635

**Published:** 2026-03-30

**Authors:** Moritz Passenberg, Clara Guntlisbergen, Nargiz Nuruzade, Jan Best, Dieter P. Hoyer, Ulf Neumann, Hartmut Schmidt, Katharina Willuweit, Georgios Konstantis, Jassin Rashidi-Alavijeh

**Affiliations:** 1 Department of Gastroenterology, Hepatology and Transplant Medicine, Medical Faculty, University of Duisburg-Essen, 45147 Essen, Germany; moritz.passenberg@uk-essen.de (M.P.);; 2 Department of General, Visceral and Transplantation Surgery, Medical Faculty, University of Duisburg-Essen, 45147 Essen, Germany

**Keywords:** acute on chronic liver failure, liver transplantation, non-acute decompensation, ultrasound examination

## Abstract

**Background and Aims:** Ascites is a common complication of cirrhosis. Its presence is associated with poorer outcomes. This study evaluates the association between ascites burden, including absolute thickness and longitudinal changes as a surrogate parameter of ascites volume and dynamics, and transplant-free survival in patients with cirrhosis. **Methods:** In this prospective single-center study, patients with cirrhosis underwent blood analysis, physical examination, and standardized ultrasound assessment of ascites at inclusion and after 3 months. Ascites burden was assessed by measuring ascites thickness in predefined abdominal compartments, which served as a surrogate for ascites volume. A 12-month follow-up recorded clinical progress, hospitalizations, decompensation, liver failure, transplantation, and death. **Results:** A total of 272 patients with cirrhosis (males 60%, median age 56 years, median MELD-Na 14) were included. Among them, 40 (15%) died or underwent liver transplantation during follow-up. These patients had significantly greater ascites thickness in all abdominal compartments at both time points as compared to survivors (all *p* < 0.001). In univariable analyses, greater baseline ascites thickness was associated with adverse outcome, with the strongest association observed for perisplenic ascites (OR 1.13, 95% CI 1.06–1.21; *p* < 0.001). In multivariate analysis, only baseline perihepatic ascites remained significantly associated with the endpoint (OR = 1.05, 95% CI [1.00–1.11], *p* = 0.045). The decrease in perisplenic ascites thickness during follow-up was in turn significantly associated with reduced risk of death or transplantation (OR 0.85, 95% CI [0.72–0.96], *p* = 0.017). **Conclusions:** In this prospective cohort, greater ascites thickness was associated with adverse outcomes in cirrhosis. Baseline perihepatic ascites and dynamic changes in perisplenic ascites emerged as potentially informative markers; however, these findings should be interpreted cautiously and require validation in larger cohorts.

## 1. Introduction

Cirrhosis is the final common pathway of different chronic liver diseases, resulting in severe complications with high morbidity and mortality due to life-threatening complications. Since liver transplantation (LT) is the only curative treatment in most cases [1,2], accurate prognostic assessment is essential for optimizing timing and prioritization of transplant listing, particularly when taking into account organ shortage and high waiting list mortality [1,3].

Ascites represents a landmark in the natural course of cirrhosis, often representing the transition to a decompensated stage with severe deterioration of prognosis [4]. Its onset is associated not only with reduced quality of life but also with increased risks of infections, renal dysfunction, and acute-on-chronic liver failure (ACLF) [5,6]. For this reason, the presence and severity of ascites are also part of established prognostic models, such as the Child–Pugh Score (CPS) [7].

Although the prognostic relevance of ascites presence is well established, the relevance of the quantitative extent of ascites and its dynamic changes over time has not yet been sufficiently evaluated. Current grading systems, such as that of the International Club of Ascites (ICA) [8], are based on semi-quantitative and strongly examiner-dependent criteria, limiting their objectivity and prognostic utility.

In this context, the concept of non-acute decompensation (NAD), which includes the presence of mild ascites, has gained increasing attention as a potential marker of disease progression [9,10,11]. However, the prognostic relevance of NAD remains poorly understood. A more objective, ultrasound-based quantitative assessment of ascites, particularly in the outpatient setting, could enhance early risk stratification and inform transplant decision making.

For the depicted reasons, we aimed to study (a) the association between ultrasound-assessed ascites burden and transplant-free survival, and (b) whether longitudinal changes in ascites burden are independently associated with adverse outcomes in patients with cirrhosis. Our aim is to contribute to a more objective and dynamic approach to risk stratification in advanced liver disease, in particular with regard to prioritization of transplantation.

## 2. Patients and Methods

### 2.1. Study Design

For this prospective, single-center analysis, data were collected and analysed from 272 patients with cirrhosis who were treated in the LT outpatient unit of University Hospital Essen. Patients were enrolled between March and October 2023. Eligible patients were required to be at least 18 years of age, to have confirmed cirrhosis based on prior histology, imaging findings consistent with cirrhosis, and/or documented hepatology records, and to attend the outpatient clinic for routine follow-up during the recruitment period. Patients with HIV infection, prior LT, and pregnancy were excluded. No further exclusion criteria were applied. At study inclusion, all patients were managed in an outpatient setting. Thus, the cohort represents an outpatient-managed cirrhosis population and was not intended to include patients presenting during hospitalization for acute decompensation or ACLF. No formal a priori sample size calculation was performed, as the cohort comprised consecutively included eligible patients during the predefined recruitment period.

Upon inclusion, patients underwent blood analysis, physical examination, and standardized ultrasound assessment of ascites, as described below. Examinations were repeated after three months. Data on medications, preexisting conditions, hepatic complications, decompensation events, and liver failure were collected. A 12-month follow-up assessed clinical progress, hospitalizations, decompensation, liver failure, LT, or death. The primary outcome of the main regression analyses was the composite endpoint of LT or death within 12 months after study inclusion. In addition, time-to-event analyses were performed for the combined endpoint of death/LT. To specifically assess death while accounting for LT as a competing event, competing-risk analyses were additionally conducted.

Ascites measurements using ultrasound were performed by a collective of three trained examiners using a standardized protocol. Ascites burden was operationalized by standardized ultrasound measurements of ascites thickness in predefined abdominal compartments, which were used as surrogate markers of ascites volume and its dynamic changes over time. To ensure consistency, examiners received standardized training and procedures were carefully performed. Inter-observer variability was not formally assessed and therefore remains a limitation. Ascites thickness was measured in millimeters at three predefined anatomical sites:

- Perihepatic: between the 9th and 10th intercostal space, between the midaxillary and midclavicular lines;
- Perisplenic: same intercostal level and orientation as perihepatic;
- Pelvic: in the lower abdomen above the symphysis.

Ascites was also categorized according to the ICA classification as grade 0 (no ascites), grade 1 (mild ascites detectable only by ultrasound), grade 2 (moderate ascites), or grade 3 (large/gross ascites) and was recorded as a clinical reference measure [8].

### 2.2. Patient Cohort

Demographic, clinical, and laboratory data were obtained at baseline and follow-up visits from the outpatient visits and from the medical records. Demographics included age, sex, and body mass index (BMI). The etiology of cirrhosis and associated complications was included. Laboratory values included leukocytes, haemoglobin, platelets, INR, quick, aPTT, fibrinogen, sodium, potassium, AST, ALT, GGT, AP, albumin, serum creatinine, and total bilirubin. Information on medications used (diuretic, non-selective beta-blockers, rifaximin) was also collected.

The study was conducted in accordance with the Helsinki Declaration of 1975 and was approved by the local ethics committee of the University Hospital Essen, Germany (23-11088-BO). Informed consent was obtained in writing from all patients.

### 2.3. Statistical Analysis

Categorical variables were reported as absolute numbers and percentages. Continuous variables were summarized as means with standard deviations (SDs) or as medians with interquartile ranges (IQRs), depending on data distribution. Normality was assessed using the Shapiro–Wilk test; a *p*-value < 0.05 was considered indicative of non-normal distribution.

To compare transplant-free survivors with patients who either underwent LT or died (LT/death), unpaired Student’s *t*-tests were used for normally distributed variables and Mann–Whitney *U* tests for non-normally distributed variables. For categorical variables, comparisons were performed using the chi-square test with Pearson approximation or Fisher’s exact test when expected frequencies were below five.

Delta ascites (Δ-ascites, change in ascites thickness) in the perihepatic, perisplenic, and pelvic compartments was calculated as the baseline minus follow-up (second visit) measurement. Thus, positive values indicate a decrease and negative values an increase in ascites thickness over time.

Logistic regression models were further used to assess the association between quantitative ascites measurements and the composite endpoint of LT or death. Univariate logistic regression analyses were first performed for each ascites measurement at baseline and follow-up to characterize unadjusted associations. Multivariable logistic regression models were subsequently fitted to estimate the adjusted association between each ascites parameter and the composite endpoint. These analyses were not designed as a data-driven model-building exercise and were not intended for the development of a prognostic prediction model. Rather, they represent an adjusted regression framework aimed at evaluating whether specific ascites measurements were independently associated with outcome after accounting for established clinical confounders. Covariates were selected a priori based on clinical relevance and their established role as potential confounders of the association between ascites burden and outcome in patients with cirrhosis: TIPS placement, changes in diuretic therapy, paracentesis at follow-up, MELD-Na score, age, and BMI. No univariable significance threshold was applied as a criterion for covariate inclusion. Discriminatory performance was evaluated using the area under the receiver operating characteristic curve (AUC) with 95% confidence intervals calculated via the DeLong method. The optimal classification threshold was identified by maximizing Youden’s index. Confusion matrix metrics, including accuracy, sensitivity, specificity, positive predictive value (PPV), negative predictive value (NPV), and F1 score, were reported for each model. These measures are presented as descriptive model characteristics and should not be interpreted as evidence of clinical prediction performance.

To assess internal validity and reduce the risk of overfitting, two complementary approaches were applied. First, bootstrap validation of the multivariable logistic regression models was performed using 500 resampling iterations, providing optimism-corrected estimates of discrimination (AUC), calibration (calibration slope and intercept), and overall fit (Brier score). Second, as a sensitivity analysis, penalized logistic regression using the least absolute shrinkage and selection operator (LASSO) was applied, with each ascites parameter evaluated as the primary predictor within the same covariate framework. The penalty parameter (λ) was optimised via 10-fold cross-validation, and all predictors were standardised prior to model fitting to ensure comparability of coefficients across predictors.

To further characterize the prognostic relevance of ascites dynamics, patients were dichotomised according to the change in sonographically measured ascites between baseline (T1) and follow-up (T2) using any increase in ascites (T2 > T1).

Transplant-free survival for the combined endpoint of death/LT was estimated over the 12-month follow-up period (defined as 52 weeks for time-to-event analyses). Group differences were assessed with the log-rank test. To account for the competing risk of LT, cumulative incidence functions (CIF) were additionally estimated using the Aalen-Johansen estimator and compared with Gray’s test. The association between ascites progression and the subdistribution hazard of death was quantified using the Fine-Gray proportional subdistribution hazards model, expressed as subdistribution hazard ratios (sHRa) with 95% confidence intervals. In all competing-risk analyses, LT was coded as the competing event (status 2), death as the primary event (status 1), and patients without an event at 52 weeks as censored (status 0).

All statistical analyses were conducted in R (version 4.5.2) using the packages survival, survminer, cmprsk, glmnet, boot, and pROC. A two-sided *p*-value < 0.05 was considered statistically significant.

## 3. Results

### 3.1. Patient Characteristics

A total of 272 patients with cirrhosis were included in the study (Table 1). All patients underwent standardized ultrasound examinations at baseline and at the 3-month follow-up visit. The median age was 56 years (IQR 47.8–62), and the majority were male (*n* = 163, 60%). During the 12-month follow-up period, 40 patients (15%) either died (n = 20, 7.5%) or underwent LT (n = 20, 7.5%).

Patients who reached the composite endpoint had significantly more advanced liver disease. Higher CPS classes and elevated MELD-Na scores were strongly associated with death or LT (median MELD-Na: 18 vs. 11; *p* < 0.001). Similarly, ALBI scores were significantly worse in this group (−1.66 vs. −2.33; *p* < 0.001). According to the ICA classification, a greater proportion of patients who died or underwent LT had moderate to severe ascites. Higher ICA grades were significantly associated with adverse outcomes (*p* < 0.001).

Markers of liver function were markedly worse among patients who reached the endpoint (Table 2). These individuals had higher INR and bilirubin levels and lower albumin concentrations (*p* < 0.001 for all). Kidney function was worse in the group of patients who died or received LT, as indicated by significantly higher serum creatinine levels (1.23 vs. 0.98 mg/dL; *p* = 0.013).

Systemic inflammation was more pronounced in patients who died or received LT, with significantly elevated serum IL-6 levels (median 6.4 vs. 3.9 pg/mL; *p* < 0.001). These patients also had significantly lower hemoglobin levels (10.96 vs. 12.33 g/dL; *p* < 0.001), along with higher ferritin concentrations and transferrin saturation (*p* = 0.004 and *p* = 0.027, respectively). Transient elastography revealed significantly higher liver stiffness in the event group (mean 58.3 vs. 30.1 kPa; *p* < 0.001), whereas CAP values did not differ significantly between groups (*p* = 0.541). There were no significant differences between groups regarding splenomegaly, hepatic encephalopathy, TIPS placement during follow-up, or BMI.

Table 1 and Table 2 summarize baseline patient characteristics and laboratory parameters. Three-month follow-up data are provided in Supplementary Tables S1 and S2.

### 3.2. Association Between Ascites Burden and Outcome

**Figure 1:**
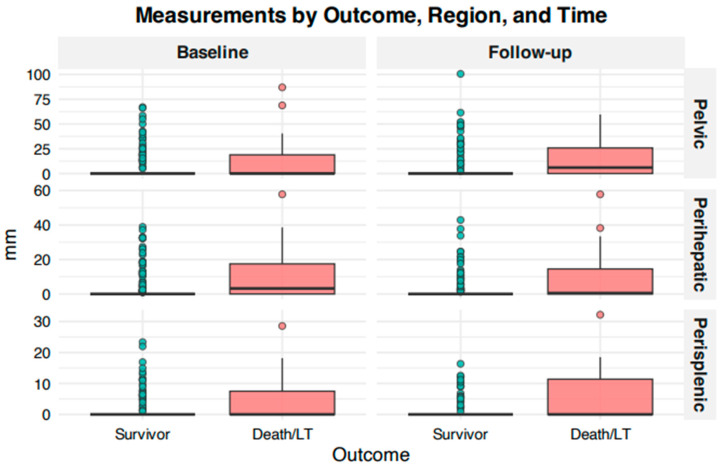
Baseline and Follow-up boxplot.

Patients who died or underwent LT (n = 40) exhibited significantly greater ascitic thickness in all compartments at both time points compared to those who survived without LT (n = 232) (Table 3). At baseline, mean ascitic thickness in the pelvic compartment was 11.83 mm (SD 19.79) in the death/LT group versus 4.06 mm (SD 11.61) in transplant-free survivors (*p* < 0.001). Corresponding values in the perihepatic compartment were 10.23 mm (SD 14.09) versus 2.77 mm (SD 7.34), respectively (*p* < 0.001), and in the perisplenic compartment 4.38 mm (SD 6.88) versus 1.07 mm (SD 3.47), respectively (*p* < 0.001) (Figure 1).

Univariable logistic regression analyses demonstrated a significant association between greater ascites thickness and the composite endpoint (death/LT) in all three compartments. The strongest association was observed for perisplenic ascites (OR 1.13, 95% CI 1.06–1.21, *p* < 0.001), followed by perihepatic ascites (OR 1.07, 95% CI 1.04–1.10, *p* < 0.001), and pelvic ascites (OR 1.03, 95% CI 1.01–1.05, *p* = 0.002) (Table 4). Discriminative performance, as indicated by AUC values, ranged from 0.63 (pelvic) to 0.69 (perihepatic) (Supplementary Figure S1).

In multivariable logistic regression analyses adjusted for clinical covariates (TIPS placement, changes in diuretic medication, paracentesis at follow-up, MELD-Na score, age, and BMI), baseline perihepatic ascites remained independently associated with the combined endpoint of death or LT in adjusted analyses (OR = 1.05, 95% CI [1.00–1.11], *p* = 0.045). Baseline perisplenic (*p* = 0.20) and pelvic (*p* = 0.94) ascites did not retain significance (Supplementary Table S3).

### 3.3. Association of Ascites Dynamics (Δ-Ascites) with Adverse Outcomes

**Figure 2:**
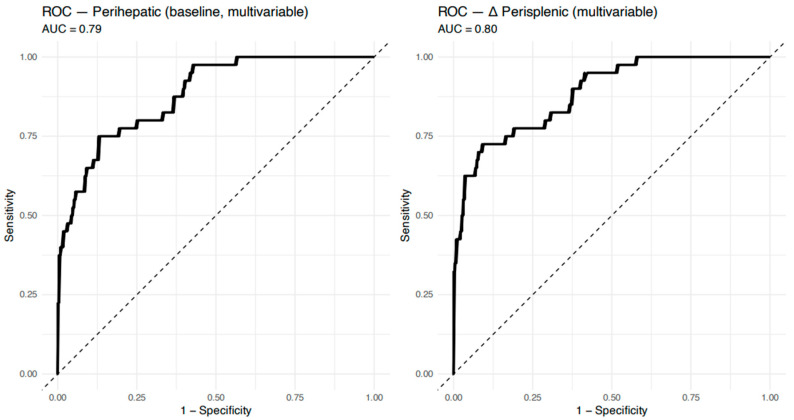
ROC curves for multivariable logistic regression.

Regarding dynamic changes in multivariable analysis, only perisplenic Δ-ascites remained significantly associated with the endpoint in adjusted analyses (OR = 0.84, 95% CI [0.72–0.96], *p* = 0.017) (Supplementary Figure S2). Changes in the perihepatic and pelvic compartments were not significantly associated with the endpoint (both *p* > 0.05) (Supplementary Table S4; Supplementary Figure S3). Descriptive discrimination measures of the adjusted models yielded AUC values ranging from 0.79 to 0.80 (Figure 2). Among the adjusted models, the model including baseline perihepatic ascites achieved the highest overall accuracy (87%) and F1 score (0.59). Negative predictive values were uniformly high across all models (range: 0.94–0.95), although these measures should be interpreted descriptively rather than as evidence of clinical prediction performance. A summary of the adjusted odds ratios, 95% confidence intervals, and *p*-values for all variables is provided in Table 5.

### 3.4. Sensitivity Analysis: Penalized Regression

In bootstrap-validated multivariable models, discrimination was moderate (AUC ≈ 0.74–0.75 after optimism correction) with acceptable Brier scores. Calibration slopes showed wide uncertainty intervals, indicating potential variability in predicted risks. In LASSO-penalized models, discrimination was comparable (AUC 0.78–0.79) with slightly higher accuracy and preserved specificity and NPV, though sensitivity and PPV remained modest. In bootstrap-validated and penalized sensitivity analyses, the overall pattern and direction of associations were broadly consistent with the main analyses.

The results from the LASSO analysis and bootstrap validation can be seen in Supplementary Tables S5–S7 and Supplementary Figures S5–S7.

### 3.5. Time-to-Event and Competing-Risk Analyses

Kaplan–Meier analyses for the combined endpoint of death/LT within 52 weeks demonstrated significantly lower event-free survival in patients with any increase in ascites between baseline and follow-up. The strongest effect was observed for perisplenic ascites (log-rank *p* < 0.0001), followed by pelvic ascites (*p* = 0.00011), whereas the association for perihepatic ascites was weaker but remained statistically significant (*p* = 0.041) (Supplementary Figure S7).

In separate competing-risk analyses, with LT treated as the competing event, any increase in ascites from T1 to T2 was consistently associated with a higher subdistribution hazard for death across all three anatomical compartments. Specifically, any increase in pelvic ascites was associated with a more than fivefold higher risk of death (sHR 5.55, 95% CI 2.23–13.83; *p* < 0.001), while similar associations were also observed for perihepatic ascites (sHR 4.05, 95% CI 1.61–10.18; *p* = 0.003) and perisplenic ascites (sHR 5.49, 95% CI 2.08–14.49; *p* < 0.001) (Table 6, Supplementary Figure S8).

Together, these time-to-event analyses support the association between worsening ascites over time and adverse clinical outcomes.

## 4. Discussion

This prospective study suggests an association between ultrasound-assessed ascites burden and transplant-free survival in a cohort of 272 patients with cirrhosis. In a well-characterized outpatient cohort, greater baseline ascites thickness was associated with an increased risk of death or LT across all abdominal compartments, although only perihepatic ascites remained significantly associated with the composite endpoint in multivariable analysis. Furthermore, longitudinal changes in ascites—particularly in the perisplenic compartment—were associated with outcome over time. This was further supported by time-to-event analyses. For the combined endpoint of death or LT, Kaplan–Meier analyses showed lower event-free survival in patients with increasing ascites over time. In addition, competing-risk analyses demonstrated that increasing ascites was associated with a higher subdistribution hazard of death when LT was treated as a competing event. These findings highlight the potential clinical relevance of dynamic ultrasound-based monitoring.

The development of ascites marks a pivotal event in the natural history of cirrhosis, with portal hypertension being the primary driver of ascites formation. It is well established that the presence or new onset of ascites in patients with cirrhosis is associated with a poorer prognosis and generally reflects a deterioration of liver function as a consequence of progressive portal hypertension [11,12,13]. The ICA consensus conference in 2003 emphasized the strong association between the occurrence of ascites and adverse clinical outcomes [5]. Moreover, even small amounts of ascites appear to have prognostic relevance and have gained increasing attention within the framework of NAD as a distinct pathway of disease progression [11]. In this context, Tonon et al. demonstrated that the isolated presence of grade 1 ascites is associated with a significantly worse prognosis compared to cirrhotic patients without ascites in an outpatient cohort [14]. Our results build on these observations by quantifying ascites burden and suggest that even moderate increases in ascites thickness may be associated with clinical endpoints. These findings support recent concepts such as NAD and further suggest that dynamic changes in ascites thickness over time may provide additional prognostic information.

Beyond the mechanical and hemodynamic factors, ascites may also reflect immune dysregulation, which is recognized as a key driver in cirrhosis [15,16]. In line with this concept, we observed higher IL-6 levels in patients with adverse outcomes. This observation supports a possible link between ascites burden and systemic immune activation [17,18].

The compartment-specific differences observed in our study raise interesting clinical and pathophysiological questions. While perihepatic ascites was the strongest baseline correlate of the composite endpoint, only perisplenic ascites dynamics were associated with outcome over time. This may reflect local anatomical or physiological differences in fluid accumulation and resorption. For example, perihepatic ascites may be more static or influenced by confounding factors (e.g., paracentesis, diaphragmatic motion), whereas perisplenic ascites might be more sensitive to subtle volume shifts. In this context, it is noteworthy that splenic alterations, such as enlargement or increased stiffness, are involved in the pathophysiology of portal hypertension and may also contribute to ascites formation [19,20]. Therefore, it is conceivable that changes in the left upper abdominal compartment (perisplenic region) may be more strongly associated with the underlying hemodynamic consequences of portal hypertension. However, direct evidence supporting this interpretation is currently not available, and further investigation in larger cohorts is needed to clarify these mechanisms.

Our study has several limitations. First, this was a single-center study conducted in an outpatient liver transplant clinic, which may limit generalizability and may have introduced the possibility of selection bias. Second, despite standardized ultrasound performed by a limited number of trained examiners, variability due to patient positioning, body habitus, or anatomical variation may still occur. Inter-observer variability was not formally quantified. Third, despite multivariable adjustment, residual confounding remains likely, particularly with regard to disease severity, treatment changes, and interventions such as paracentesis. Moreover, spleen stiffness was not assessed, which has been suggested as a potential predictor of ascites. Finally, the limited number of clinical events restricts statistical robustness and may increase the risk of model instability or overfitting despite internal validation procedures. Accordingly, our findings should be interpreted as exploratory and hypothesis-generating rather than definitive evidence of independent prognostic value. Nevertheless, the observational design reflects real-world clinical practice.

In summary, our findings suggest that both baseline ascites burden and longitudinal ascites dynamics may be associated with adverse outcomes in patients with cirrhosis. Baseline perihepatic ascites thickness and longitudinal changes in perisplenic ascites emerged as potentially informative markers of adverse outcome in this outpatient cohort. However, given the study limitations, these findings should be interpreted cautiously. Future, preferably multicenter prospective studies with larger cohorts are needed to validate whether compartment-specific ultrasound assessment of ascites provides robust incremental prognostic value beyond established clinical predictors.

## Figures and Tables

**Table 1:** Patient characteristics at baseline.

	Death/LT n = 40	Survivor n = 232	*p*-Value
Sex (male), n (%)	31 (77.5)	132 (56.9)	0.015
Listed, n (%)	29 (72.5)	199 (85.8)	0.059
Age (years), median (IQR)	57.5 [49.75, 62.5]	56 [47.75, 62]	0.653
BMI (kg/m^2^), median (IQR)	25.55 [22.32, 29.73]	26.35 [23.1, 30.9]	0.486
MELD-Na, median (IQR)	16 [12.75, 19.5]	11 [9, 13]	**<0.001**
**ICA Grade**			**<0.001**
ICA Grade 0, n (%)	15 (37.5)	177 (76.3)	
ICA Grade 1, n (%)	16 (40)	37 (15.9)	
ICA Grade 2, n (%)	3 (7.5)	8 (3.4)	
ICA Grade 3, n (%)	6 (15)	10 (4.3)	
Hepatic encephalopathy, n (%)	1 (2.5)	0 (0)	0.147
Splenomegaly, n (%)	37 (92.6)	215 (92.7)	1.000
Torasemide, n (%)	22 (55)	92 (39.7)	0.219
Furosemide, n (%)	4 (10)	24 (10.3)	1.000
Spironolactone, n (%)	22 (55)	131 (56.5)	0.884
Rifaximin, n (%)	18 (45)	62 (26.7)	0.049
NSBB, n (%)	33 (82.5)	162 (69.8)	0.257
Metformin, n (%)	3 (7.5)	9 (3.9)	0.483
Renal insufficiency, n (%)	3 (7.5)	17 (7.3)	1.000
Vitamin D supplementation, n (%)	16 (40)	77 (33.2)	0.549
Iron supplementation, n (%)	6 (15)	35 (15.1)	1.000
Vitamin B12 supplementation, n (%)	1 (2.5)	2 (0.9)	0.473
Folic acid supplementation, n (%)	11 (27.5)	34 (14.7)	0.125
**Child–Pugh score**			**<0.001**
CHILD–PUGH A, n (%)	13 (32.5)	161 (69.4)	
CHILD–PUGH B, n (%)	21 (52.5)	63 (27.2)	
CHILD–PUGH C, n (%)	6 (15)	1 (0.4)	
TIPS since inclusion	1 (2.5)	4 (1.7)	0.553
Liver stiffness (kPa), mean (SD)	54.63 [19.94]	37.56 [26.09]	**<0.001**
CAP (dB/m), mean (SD)	252.79 [72.43]	248.05 [82.61]	0.541
**Etiology of cirrhosis**			0.796
MASH, n (%)	2 (5)	18 (7.6)	
Alcohol, n (%)	17 (42.5)	106 (45.7)	
Viral, n (%)	4 (10)	22 (9.5)	
Autoimmune, n (%)	9 (22.5)	50 (21.6)	
Hereditary liver diseases, n (%)	0 (0)	5 (2.6)	
Other, n (%)	8 (20)	31 (13.4)	

Categorical data are given as absolute numbers and percentages (%). Comparisons between groups were performed by an χ^2^-test with Pearson approximation or Fisher’s exact test. Numeric data are presented as mean and SD or median (IQR). Statistical significance was assessed by using the Mann–Whitney U test. A value of *p* ≤ 0.05 was assumed to be significant. Bold values represent statistical significance. BMI: body mass index; ICA: International Club of Ascites; LT: liver transplantation; MELD-Na: model of end-stage liver disease; MASH: metabolic-associated steatotic hepatitis; NSBB: non-selective beta blocker; TIPS: transjugular intrahepatic portosystemic shunt. The bold formatting was used to emphasize the statistical significance of the *p*-values.

**Table 2:** Laboratory parameters at baseline.

	Death/LT Median [IQR]	Survivor Median [IQR]	*p*-Value
Leukocytes (1/nL)	5.83 [4.12, 7.31]	5.54 [4.07, 6.99]	0.469
Hemoglobin (g/dL)	11 [9.45, 12.2]	12.55 [11, 14.1]	**<0.001**
Platelets (1/fL)	100.5 [65, 127.25]	110 [70, 160]	0.204
INR	1.4 [1.19, 1.53]	1.19 [1.08, 1.31]	**<0.001**
Quick (%)	52 [45.75, 68.5]	69.5 [57, 83.25]	**<0.001**
aPTT (sec.)	30.45 [26.78, 34.75]	27.4 [25.17, 29.9]	**<0.001**
Natrium (mmol/L)	137.5 [135, 140]	140 [137, 142]	**0.011**
Potassium (mmol/L)	4.1 [3.7, 4.6]	4.2 [3.9, 4.5]	0.618
Creatinine (mg/dL)	1.03 [0.81, 1.36]	0.9 [0.72, 1.1]	**0.013**
Albumin (mg/dL)	3.25 [2.85, 3.7]	3.9 [3.4, 4.2]	**<0.001**
Total bilirubin (mg/dL)	3 [1.55, 6.05]	1.5 [0.9, 2.2]	**<0.001**
AST (U/L)	60.5 [46.25, 91.25]	42 [29.5, 59]	**<0.001**
ALT (U/L)	40.5 [29, 58.75]	32 [23, 47]	**0.034**
AP (U/L)	153.5 [111.25, 233.5]	120 [86, 179]	**0.004**
γGT (U/L)	89 [52.5, 141.5]	75 [43.5, 153.5]	0.744
Cholesterol (mg/dL)	147 [117.5, 181]	158 [130, 198]	0.120
HDL (mg/dL)	27.5 [20, 47]	48 [39, 58]	**<0.001**
LDL (mg/dL)	77 [65.5, 95.5]	97 [72, 122]	**0.028**
Triglyceride (mg/dL)	101 [79.25, 158.5]	92 [76, 128]	0.162
IL 6 (ng/L)	6.4 [4.38, 14.1]	3.9 [2.7, 5.9]	**<0.001**
Iron (µg/dL)	118 [63, 157]	106 [63.75, 140]	0.592
Ferritin (µg/L)	111 [44, 169.75]	56 [24, 115.25]	**0.004**
Transferrin (g/L)	2 [1.7, 2.48]	2.5 [2.1, 2.8]	**<0.001**
Transferrin saturation (%)	38.7 [20.92, 83.33]	30 [18.2, 44]	**0.027**
Soluble transferrin receptor (mg/L)	1.87 [1.6, 2.28]	1.66 [1.35, 2.48]	0.240
Vitamin B12 (pg/mL)	949 [744, 1232.25]	644 [471, 952]	**<0.001**
Folic acid (ng/mL)	12.98 [8.67, 19.79]	11.32 [7.84, 19.85]	0.694

Laboratory values are given as median and interquartile range (IQR). Statistical significance was determined using an unpaired Student’s *t* test assuming equal variances or Mann–Whitney U test. A value of *p* ≤ 0.05 was considered significant. Bold values represent statistical significance. ALT: alanine aminotransferase; AST: aspartate aminotransferase; AP: alkaline phosphatase; aPTT: activated partial thromboplastin time; γGT: gamma-glutamyl transferase; INR: international normalised ratio; IQR: interquartile range. The bold formatting was used to emphasize the statistical significance of the *p*-values.

**Table 3:** Univariable analysis ascites measurements between groups death/LT and survivor.

	Death/LT Mean [SD]	Survivor Mean [SD]	*p*-Value
Ascites pelvic baseline (mm)	11.83 [19.79]	4.06 [11.61]	**<0.001**
Ascites pelvic follow-up (mm)	14.09 [18.00]	3.94 [12.43]	**<0.001**
Δ-ascites pelvic (mm)	−2.27 [13.26]	0.13 [11.69]	0.219
Ascites perihepatic baseline (mm)	10.23 [14.09]	2.77 [7.34]	**<0.001**
Ascites perihepatic follow-up (mm)	8.99 [13.13]	2.01 [6.04]	**<0.001**
Δ-ascites perihepatic (mm)	1.24 [7.25]	0.76 [5.67]	0.236
Ascites perisplenic baseline (mm)	4.38 [6.88]	1.07 [3.47]	**<0.001**
Ascites perisplenic follow-up (mm)	5.21 [7.41]	0.71 [2.50]	**<0.001**
Δ-ascites perisplenic (mm)	−0.83 [3.62]	0.36 [2.81]	0.114

Values are presented as means with standard deviations (SDs). Statistical significance was determined using the Mann–Whitney *U* test depending on data distribution. A *p*-value ≤ 0.05 was considered statistically significant. Bold values indicate statistical significance. The bold formatting was used to emphasize the statistical significance of the *p*-values.

**Table 4:** Univariable logistic regression death/LT after 12 months.

	OR	CI (95%)	AUC	*p*-Value
Ascites pelvic baseline (mm)	1.03	1.01–1.05	0.63	**0.002**
Δ-ascites pelvic (mm)	0.983	0.955–1.01	0.55	0.238
Ascites perihepatic baseline (mm)	1.07	1.04–1.10	0.69	**<0.001**
Δ-ascites perihepatic (mm)	1.01	0.956–1.07	0.55	0.635
Ascites perisplenic baseline (mm)	1.13	1.06–1.21	0.64	**<0.001**
Δ-ascites perisplenic (mm)	0.845	0.735–0.963	0.55	**0.013**

Values are presented as odds ratios (ORs) with 95% confidence intervals (CIs). Model performance was evaluated using the area under the receiver operating characteristic curve (AUC). Statistical significance was determined using univariable logistic regression analysis. A *p*-value ≤ 0.05 was considered statistically significant. The bold formatting was used to emphasize the statistical significance of the *p*-values.

**Table 5:** Adjusted metrics of ascites measurements for death/LT at 12 months.

Model	OR (95% CI)	*p*-Value	AUC	Sensitivity	Specificity	Accuracy	PPV	NPV	F1
Ascites pelvic (mm)	1.00 (0.96–1.04)	0.94	0.789	0.72	0.79	0.78	0.36	0.95	0.48
Ascites perihepatic (mm)	1.05 (1.00–1.11)	**0.045**	0.794	0.67	0.90	0.87	0.52	0.94	0.59
Ascites perisplenic (mm)	1.07 (0.97–1.18)	0.20	0.789	0.64	0.86	0.83	0.43	0.94	0.52
Δ-ascites pelvic	0.97 (0.95–1.00)	0.057	0.801	0.67	0.87	0.84	0.45	0.94	0.54
Δ-ascites perihepatic	0.98 (0.92–1.04)	0.46	0.789	0.75	0.74	0.74	0.33	0.95	0.45
Δ-ascites perisplenic	0.84 (0.72–0.96)	**0.017**	0.802	0.64	0.88	0.85	0.47	0.94	0.54

Values are presented as adjusted odds ratios (aORs) with 95% confidence intervals (CIs). Model performance was assessed using area under the receiver operating characteristic curve (AUC), sensitivity, specificity, accuracy, positive predictive value (PPV), negative predictive value (NPV), and F1 score. Statistical significance was determined using adjusted logistic regression analysis. A *p*-value ≤ 0.05 was considered statistically significant. The bold formatting was used to emphasize the statistical significance of the *p*-values.

**Table 6:** Fine–Gray competing-risk regression for death according to any increase in ascites.

Location	Contrast	sHR	95% CI	*p*-Value
Pelvic	Any vs. no increase in ascites	5.55	2.23–13.83	<0.001
Perihepatic	Any vs. no increase in ascites	4.05	1.61–10.18	0.003
Perisplenic	Any vs. no increase in ascites	5.49	2.08–14.49	<0.001

sHR: subdistribution hazard ratio; CI: confidence interval. LT was treated as the competing event.

## Data Availability

The data are not publicly available owing to ethical restrictions regarding patients’ data, but are available upon reasonable request from the corresponding author.

## Supplementary Materials

The following supporting information can be downloaded at: https://www.mdpi.com/article/10.3390/jcm15072635/s1, Figure S1: ROC Curves univariable logistic regression; Figure S2: Forest plot—1 year transplant free survival; Figure S3: Δ-ascites Boxplots; Figure S4: Calibration Plots; Figure S5: AUC Bootstrap; Figure S6: Brier Bootstrap; Figure S7: Kaplan-Meier Curves Ascites increase; Figure S8: Competing Risk plots; Table S1: Patient characteristics at three-month follow-up; Table S2: Laboratory parameters at three-month follow-up; Table S3: Multivariable logistic regression death/LT after 12 months; Table S4: Multivariable analysis logistic regression of ascites dynamics with death/LT after 12 months; Table S5: LASSO analysis; Table S6: LASSO Bootstrap ascites perihepatic baseline and ascites perisplenic baseline; Table S7: Bootstrap validation of classical multivariable logistic regression models.

## Abbreviations

ACLF	Acute on chronic liver failure
ALT	Alanine aminotransferase
AP	Alkaline phosphatase
aPTT	Activated partial thromboplastin time
AST	Aspartate aminotransferase
AUC	Area under the curve
BIC	Bayesian information criterion
BMI	Body mass index
CPS	Child–Pugh score
HBV	Hepatitis B virus
HCV	Hepatitis C virus
HDV	Hepatitis D virus
INR	International normalized ratio
IQR	Interquartile Range
LASSO	Least absolute shrinkage and selection operator
LT	Liver transplantation
MELD-Na	Model of end-stage liver disease
MASH	Metabolic-associated steatohepatitis
NAD	Non-acute decompensation
SD	Standard deviation
TIPS	Transjugular intrahepatic portosystemic shunt
γGT	Gamma-glutamyltransferase
