# Supplementary material for: Prognostic Relevance of Ascites Volume and Dynamics in Outpatients with Cirrhosis: A Prospective Cohort Study
Source: J Clin Med. 2026 Mar 30;15(7):2635. doi: 10.3390/jcm15072635 (PMC13072754; doi:10.3390/jcm15072635)
Supplement: Supplementary file 1 [file jcm-15-02635-s001.zip › jcm-4199589-supplementary.pdf]

Supplementary Material

Supplementary Figure 1: ROC Curves univariable logistic regression

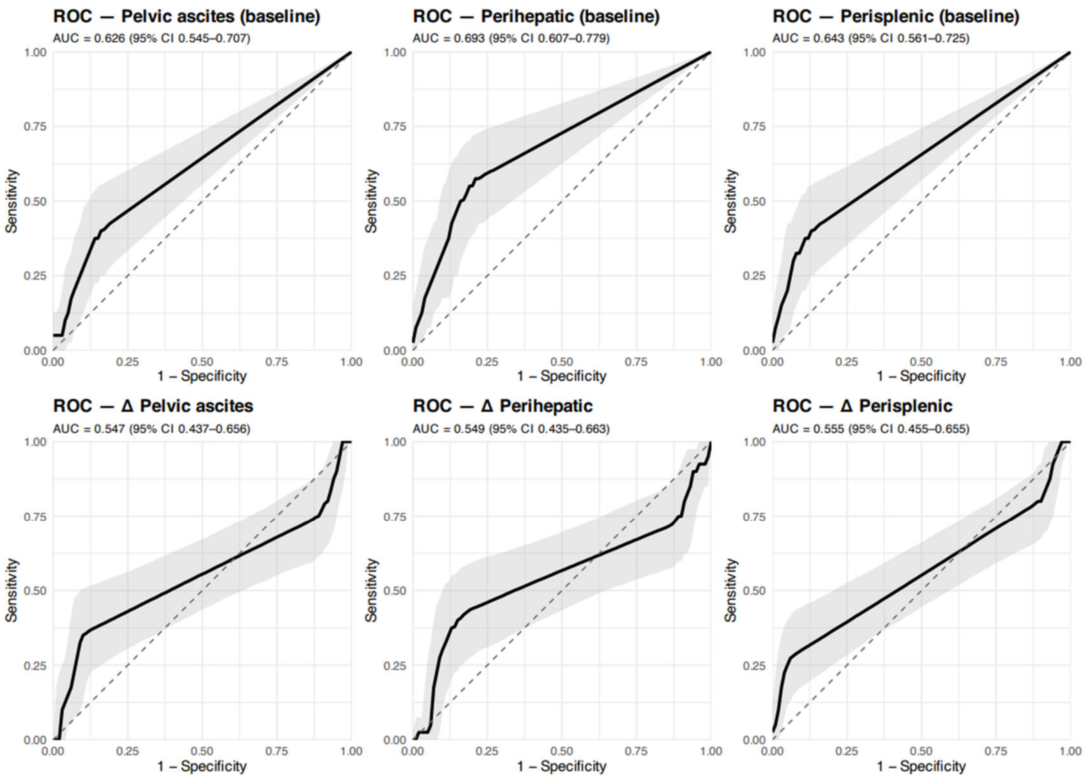

Supplementary Figure 2: Forest plot – 1 year transplant free survival

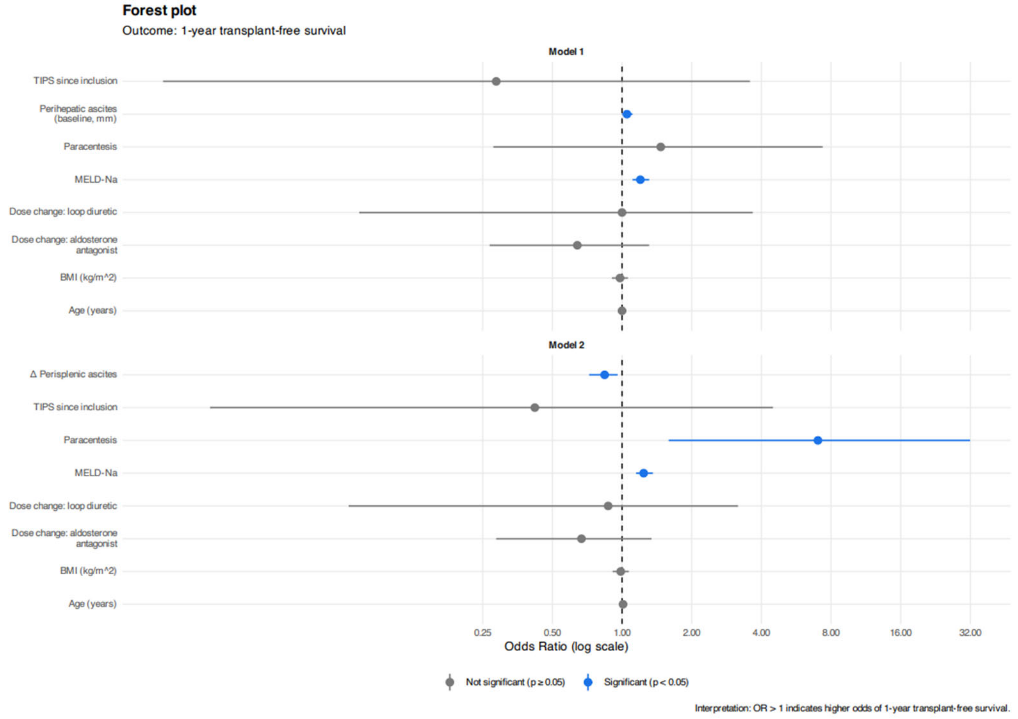

Supplementary Figure 3: Δ-ascites Boxplots

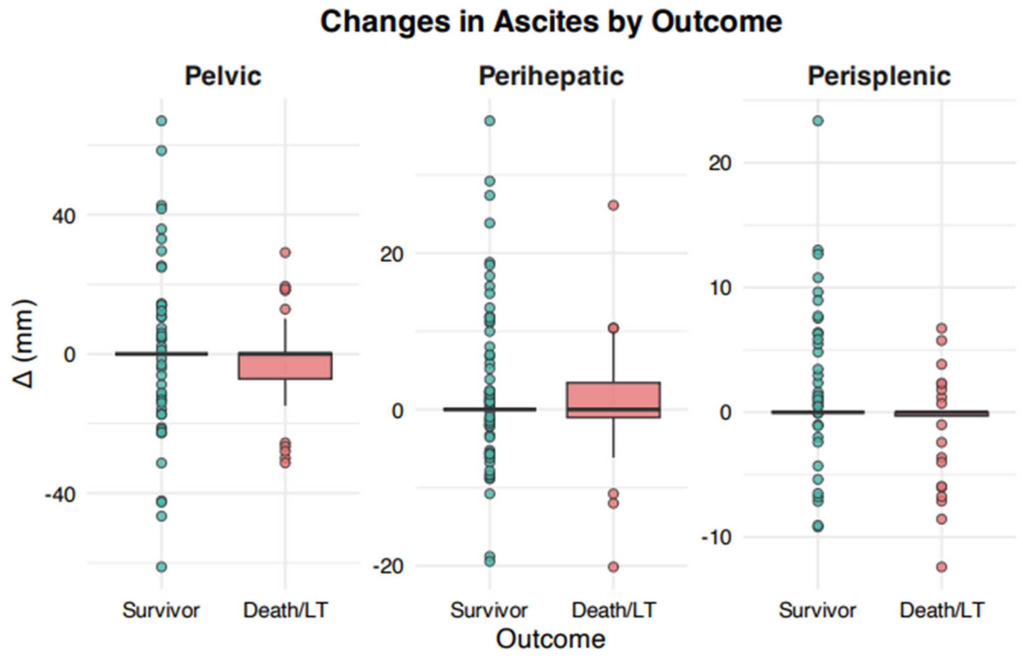

Supplementary Figure 4: Calibration Plots

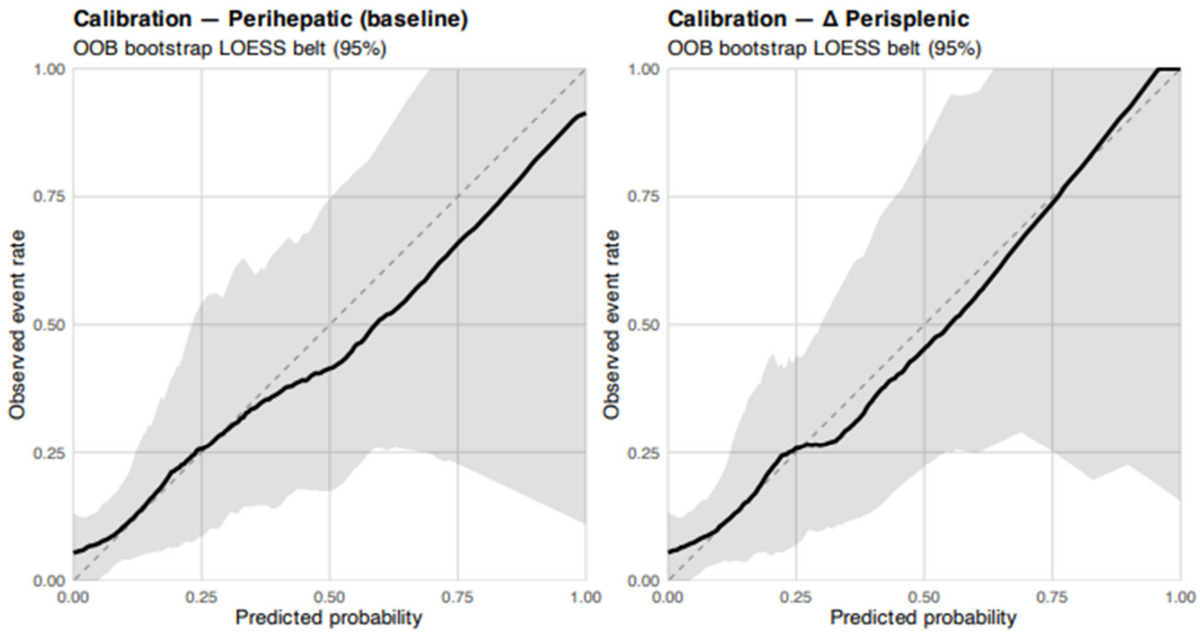

Supplementary Figure 5: AUC Bootstrap

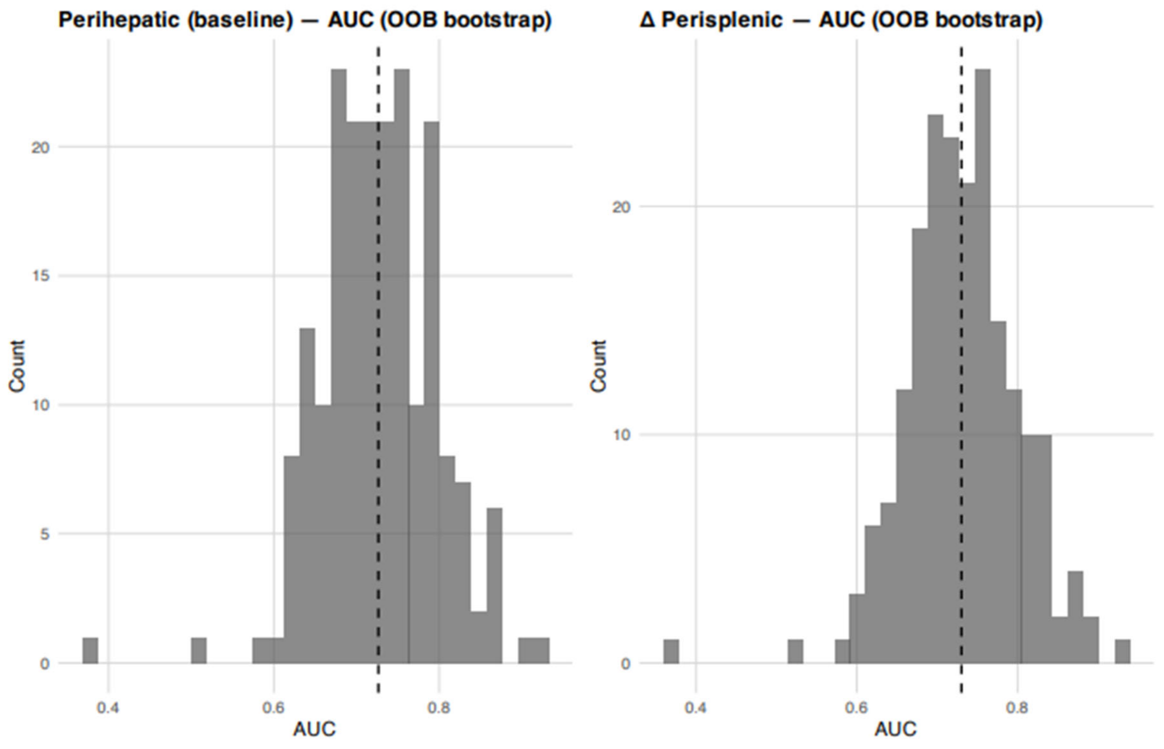

Supplementary Figure 6: Brier Bootstrap

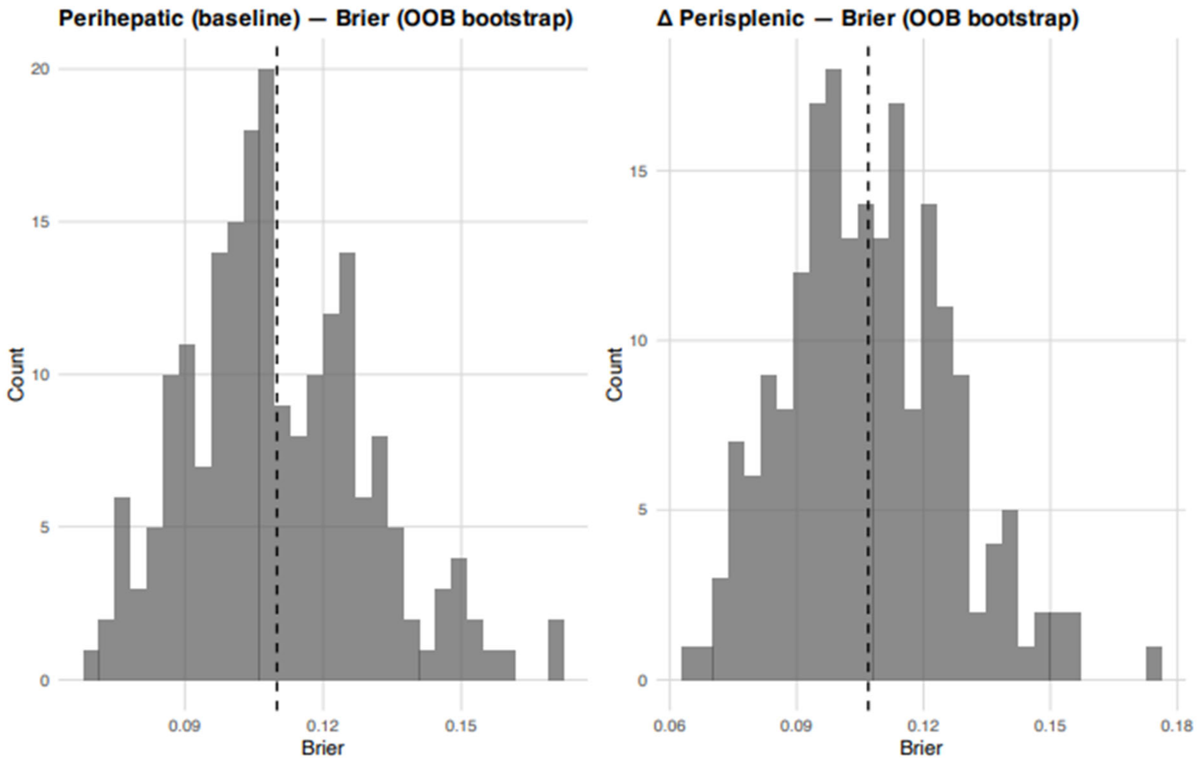

Supplementary Figure 7: Kaplan-Meier Curves Ascites increase

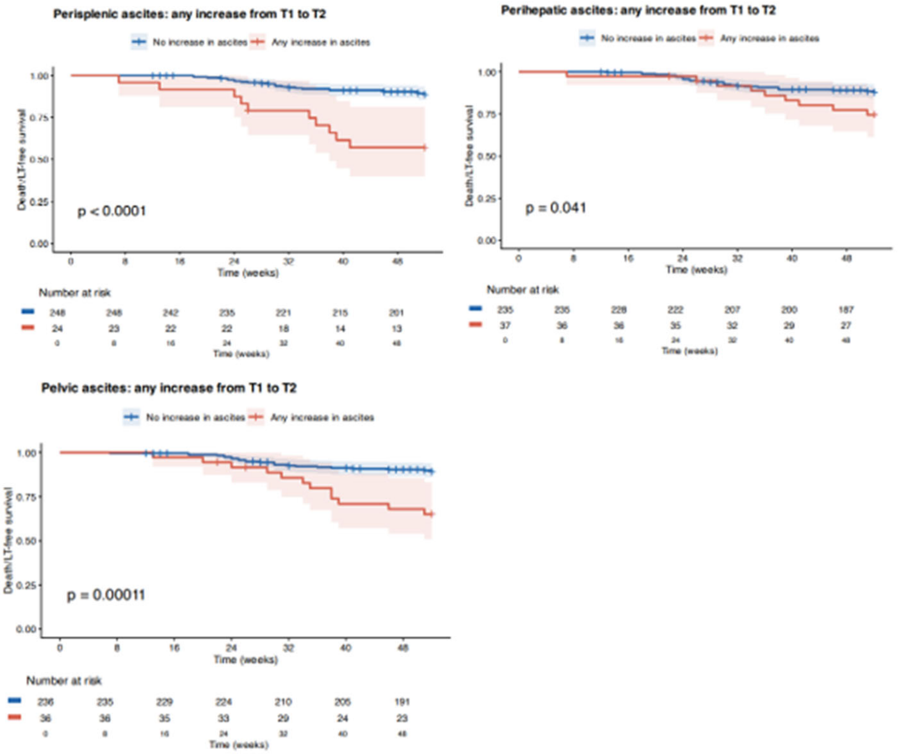

Supplementary Figure 8: Competing Risk plots

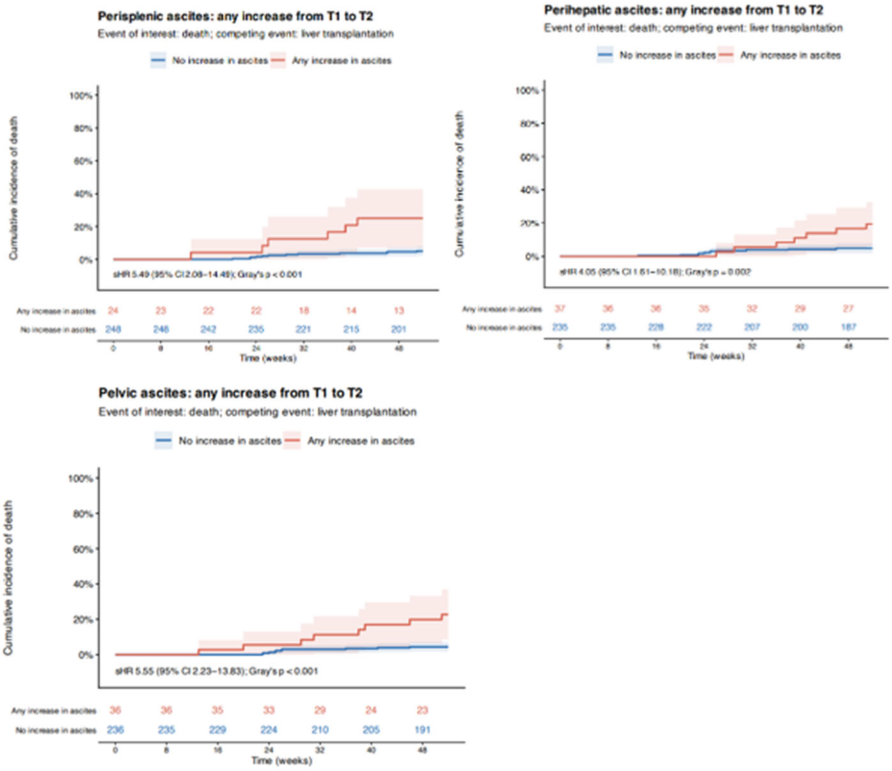

Supplementary Table 1: Patient characteristics at three-month follow-up

	**Death/LT**<br>n = 40[1]	**Survivor**<br>n = 232[1]	**P-Value[2]**
Age (years), median (IQR)	58 [50, 64]	57 [48, 63]	0.666
MELD Na, median (IQR)	16.5 [12.5, 21.0]	11.0 [9.0, 13.0]	**<0.001**
ICA Grade			**<0.001**
ICA Grade 0, n (%)	15 (38%)	183 (79%)	
ICA Grade 1, n (%)	15 (38%)	33 (14%)	
ICA Grade 2, n (%)	5 (13%)	11 (4.7%)	
ICA Grade 3, n (%)	5 (13%)	5 (2.2%)	
Hepatic encephalopathy, n (%)	6 (15%)	6 (2.6%)	**0.003**
Splenomegaly, n (%)	38 (95%)	208 (90%)	0.552
Torasemide, n (%)	24 (60%)	102 (44%)	0.086
Furosemide, n (%)	2 (5.0%)	19 (8.3%)	0.749
Spironolactone, n (%)	26 (65%)	128 (56%)	0.303
Rifaximin, n (%)	15 (38%)	61 (27%)	0.182
NSBB, n (%)	32 (80%)	167 (73%)	0.437
Metformin, n (%)	2 (5.0%)	6 (2.6%)	0.337
Renal insufficiency, n (%)	2 (5.0%)	7 (3.0%)	0.625

	Death/LT n = 40[1]	Survivor n = 232[1]	P-Value[2]
Vitamin D supplementation, n (%)	15 (38%)	89 (39%)	1.000
Iron supplementation, n (%)	5 (13%)	38 (17%)	0.644
Vitamin B12 supplementation, n (%)	1 (2.5%)	7 (3.0%)	1.000
Folic acid supplementation, n (%)	8 (20%)	33 (14%)	0.348
**Child-Pugh Score**			**<0.001**
A	9 (27%)	164 (77%)	
B	20 (61%)	48 (22%)	
C	4 (12%)	2 (0.9%)	
**TIPS since inclusion, n (%)**	1 (2.5%)	4 (1.7%)	0.553

[1] n (%); Median [Q1, Q3]

[2] Fisher's exact test; Wilcoxon rank sum test; Fisher's Exact Test for Count Data with simulated p-value (based on 2000 replicates)

Supplementary Table 2: Laboratory parameters at three-month follow-up

	**Death/LT** Median [IQR][1]	**Survivor** Median [IQR][1]	**P-value**[2]
Leukocytes (1/nl)	5.53 [4.00, 7.75]	5.10 [3.87, 6.56]	0.191
Hemoglobin (g/dL)	10.55 [8.95, 12.40]	12.80 [11.30, 14.10]	**<0.001**
Platelets (1/fl)	88 [62, 138]	106 [69, 157]	0.238
INR	1.38 [1.18, 1.64]	1.18 [1.09, 1.30]	**<0.001**

	**Death/LT** Median [IQR][1]	**Survivor** Median [IQR][1]	**P-value**[2]
Quick (%)	53 [42, 70]	69 [59, 83]	**<0.001**
aPTT (sec.)	30.5 [28.6, 35.4]	28.4 [26.2, 30.6]	**<0.001**
Natrium (mmol/L)	138.0 [135.0, 140.5]	141.0 [138.0, 142.0]	**<0.001**
Potassium (mmol/L)	4.30 [4.00, 4.70]	4.25 [3.95, 4.60]	0.400
Creatinine (mg/dL)	1.04 [0.81, 1.28]	0.92 [0.74, 1.13]	**0.041**
Albumin (mg/dL)	3.20 [2.80, 3.50]	3.80 [3.50, 4.20]	**<0.001**
Total bilirubin (mg/dL)	3.00 [1.50, 7.15]	1.40 [0.90, 2.20]	**<0.001**
AST (U/L)	48 [40, 82]	40 [27, 56]	**0.001**
ALT (U/L)	34 [24, 55]	31 [24, 44]	0.282
AP (U/L)	162 [124, 225]	121 [86, 163]	**<0.001**
γGT (U/L)	76 [45, 153]	75 [40, 141]	0.661
Cholesterine (mg/dL)	137 [106, 175]	160 [137, 190]	**0.026**
HDL (mg/dL)	31 [17, 42]	49 [41, 59]	**<0.001**
LDL (mg/dL)	72 [52, 103]	94 [74, 116]	**0.002**
Triglyceride (mg/dL)	111 [77, 159]	85 [67, 115]	**0.018**
Iron (µg/dL)	107 [70, 163]	88 [61, 123]	0.155

	**Death/LT** Median [IQR][1]	**Survivor** Median [IQR][1]	**P-value**[2]
Ferritin (µg/L)	110 [54, 283]	59 [25, 108]	**<0.001**
Transferrin (g/L)	1.85 [1.50, 2.50]	2.50 [2.10, 2.90]	**<0.001**
Transferrin saturation (%)	44 [25, 82]	26 [16, 39]	**<0.001**
Soluble transferrin receptor (mg/L)	1.85 [1.44, 2.41]	1.76 [1.37, 2.38]	0.530
Vitamin B12 (pg/mL)	942 [688, 1,204]	610 [468, 900]	**<0.001**
Folic acid (ng/mL)	17 [9, 46]	13 [9, 19]	0.252

[1] Median [Q1, Q3]

[2] Wilcoxon rank sum test

Supplementary Table 3: Multivariable logistic regression death/LT after 12 months

Model	Variable	OR	95% CI (lower)	95% CI (upper)	p-value
Ascites pelvic	Ascites pelvic baseline (mm)	1.00	0.96	1.04	0.939
	TIPS since inclusion	0.477	0.018	5.25	0.585
	Dose change furosemide	0.870	0.0665	3.11	0.867
	parecentesis	3.93	0.621	26.1	0.144
	Dose change spironolactone	0.745	0.342	1.44	0.413
	MELD-Na	1.22	1.13	1.33	**<0.001**
	Age	1.00	0.969	1.04	0.809
	BMI	0.988	0.913	1.06	0.756
Ascites perihepatic	Ascites perihepatic baseline (mm)	1.05	1.00	1.11	**0.0455**
	TIPS since inclusion	0.286	0.0104	3.57	0.374
	Dose change furosemide	1.00	0.0732	3.67	0.996
	parecentesis	1.47	0.278	7.36	0.639

Model	Variable	OR	95% CI (lower)	95% CI (upper)	p-value
	Dose change spironolactone	0.641	0.268	1.31	0.265
	MELD-Na	1.20	1.11	1.31	**<0.001**
	Age	1.00	0.968	1.04	0.896
	BMI	0.980	0.904	1.06	0.605
Ascites perisplenic	Ascites perisplenic baseline (mm)	1.07	0.97	1.18	0.200
	TIPS since inclusion	0.551	0.0227	5.73	0.648
	Dose change furosemide	0.961	0.0741	3.41	0.961
	parecentesis	1.98	0.350	10.8	0.426
	Dose change spironolactone	0.711	0.323	1.39	0.355
	MELD-Na	1.21	1.12	1.32	**<0.001**
	Age	1.00	0.967	1.04	0.911
	BMI	0.986	0.910	1.06	0.711

Supplementary Table 4: Multivariable analysis logistic regression of ascites dynamics with death/LT after 12 months

Model	Variable	OR	95% CI (lower)	95% CI (upper)	p-value
Δ-ascites pelvic	Δ-ascites pelvic	0.973	0.945	1.00	0.057
	TIPS since inclusion	1.05	0.0412	11.2	0.971
	Dose change furosemide	0.822	0.0626	3.07	0.817
	parecentesis	5.25	1.39	20.0	**0.0134**
	Dose change spironolactone	0.655	0.289	1.31	0.267
	MELD-Na	1.23	1.14	1.34	**<0.001**
	Age	1.01	0.971	1.05	0.708
	BMI	0.989	0.914	1.07	0.788
Δ-ascites perihepatic	Δ-ascites perihepatic	0.977	0.917	1.04	0.464
	TIPS since inclusion	0.601	0.0241	6.22	0.699
	Dose change furosemide	0.841	0.0650	3.04	0.835

	parecentesis	4.99	1.25	20.0	**0.0205**
	Dose change spironolactone	0.720	0.329	1.40	0.368
	MELD-Na	1.23	1.14	1.33	**<0.001**
	Age	1.00	0.969	1.04	0.818
	BMI	0.991	0.916	1.07	0.825
Δ-ascites perisplenic	Δ-ascites perisplenic	0.841	0.721	0.957	**0.017**
	TIPS since inclusion	0.420	0.0166	4.49	0.515
	Dose change furosemide	0.871	0.0659	3.17	0.869
	parecentesis	7.02	1.59	31.9	**0.0094**
	Dose change spironolactone	0.668	0.286	1.34	0.295
	MELD-Na	1.24	1.15	1.36	**<0.001**
	Age	1.01	0.971	1.05	0.696
	BMI	0.987	0.911	1.07	0.742

Supplementary Table 5: LASSO analysis

Model	OR (95% CI)	p-value	AUC	Sensitivity	Specificity	Accuracy	PPV	NPV	F1
Ascites pelvic (mm)	1.00 (0.96 – 1.04)	0.94	0.789	0.72	0.79	0.78	0.36	0.95	0.48
Ascites perihepatic (mm)	1.05 (1.00 – 1.11)	0.045	0.794	0.67	0.90	0.87	0.52	0.94	0.59
Ascites perisplenic (mm)	1.07 (0.97 – 1.18)	0.20	0.789	0.64	0.86	0.83	0.43	0.94	0.52
Δ-ascites pelvic	0.97 (0.95 – 1.00)	0.057	0.801	0.67	0.87	0.84	0.45	0.94	0.54
Δ-ascites perihepatic	0.98 (0.92 – 1.04)	0.46	0.789	0.75	0.74	0.74	0.33	0.95	0.45
Δ-ascites perisplenic	0.84 (0.72 – 0.96)	0.017	0.802	0.64	0.88	0.85	0.47	0.94	0.54

Supplementary Table 6: LASSO Bootstrap ascites perihepatic baseline and ascites perisplenic baseline

Model	AUC (95% CI)	Accuracy	Sensitivity	Specificity	PPV	NPV	F1
Perihepatic (baseline)	0.779 (0.679–0.879)	0.843	0.667	0.872	0.462	0.941	0.545
Δ Perisplenic	0.792 (0.692–0.891)	0.858	0.611	0.899	0.500	0.933	0.550

Supplementary Table 7: Bootstrap validation of classical multivariable logistic regression models

Model	AUC (apparent)	AUC (.632+)	Brier (apparent)	Brier (.632+)	AUC 95% CI	Brier 95% CI	Calibration intercept (95% CI)	Calibration slope (95% CI)
Perihepatic (baseline)	0.794	0.742	0.092	0.110	0.614–0.868	0.076–0.152	–0.847 to 0.908	–0.06 to 1.52
Δ Perisplenic	0.798	0.746	0.090	0.107	0.605–0.871	0.074–0.149	–0.838 to 0.969	–0.04 to 1.51
